# Treatment of Tardive Dyskinesia by tetrabenazine, clonazepam and vitamin E

**DOI:** 10.4103/0019-5545.49466

**Published:** 2009

**Authors:** Himanshu Sharma

**Affiliations:** Department of Psychiatry, Pramukh Swami Medical College and Shree Krishna Hospital, Karamsad – 388 325, Anand, Gujarat, India. E-mail: himanshu1969@rediffmail.com

Sir,

The term “tardive dyskinesia” has been used to refer to a group of delayed-onset abnormal involuntary movement disorders that present with rapid, repetitive, stereotypic choreoathetoid movements mostly involving the oral, buccal, and lingual areas or the trunk *(oro-bucco-lingual variety*).[1] The word *Tardiv* e means late appearing movements which start after 3months of neuroleptic exposure.[2] The annual rate is estimated at 5% for the early years, the cumulative rates over 5 years being between 20-26%.[3] Most patients with TD have schizophrenia but it can develop in-patients with depression or anxiety. Risk factors for TD are age over 40 years, affective disorder, Negroes, prolonged use and use of high potency neuroleptics in high doses, early occurrence of drug-induced Parkinson's disease, and female gender and smoking, chronic use of anticholinergic drugs and diabetes mellitus.[1,3,4] Of the various pathogenic mechanisms proposed, dopamine receptor hypersensitivity, damage to GABA containing -neurons and free radical formation from catecholamine metabolism are the most widely accepted.[5]

Following are some of the proposed guidelines for its treatment: 1) minimal dose of the causative antipsychotic or switching over to clozapine, after tapering off the offending antipsychotic drug slowly.[1] 2) Dopamine-depleting drugs -Reserpine (1 to 8 mg/day), Tetrabenazine (25 to 150 mg/day).[4,6] 3) GABA-enhancing drugs Clonazepam (1 to 4 mg/day), Valproate, Vigabatrin, Baclofen, 4) Antioxidants: Vitamin E 800 IU/bid.[1,7]

A 34-year-old male was referred to the Psychiatry OPD of our hospital with complaints of involuntary movement of orofacial region along with difficulty in breathing and swallowing, pain in nuchal region and backache. A detailed examination revealed that the patient had complex repetitive movements of orofacial region including forehead and eyebrows raising, eye closure, puckering of lips, to and fro movements of head and neck, protrusion of and twisting and darting out movement tongue in between the parted lips and repeated clenching of teeth. The patient reported that 3 years ago he had faced some financial loss as result of which he suffered from a bout of depression for which he consulted a private psychiatrist who prescribed him Trifluoperazine 10 mg along with T.Citalopram 20mg and T. Trihexyphenidyl 2mg and T. Propranolol 10-mg tid. After 2 years the patient developed TD. Patient was nonalcoholic but chronic smoker and tobacco chewer since 15 years.

His baseline assessment of abnormal involuntary movement scale (AIMS)[8] revealed a score of 21 qualifying for diagnosis of Tardive Dyskinesia (TD). All psychotropic drugs were tapered off and a course of Tetrabenazine 25mg ½ hs bid was started which was increased to 25mg bid and then tid after 2 weeks. In addition to this Clonazepam 0.5 mg I hs and Cap Vit E 400 mg 1 OD were added. The patient reported a dramatic improvement within first 2 weeks of starting the treatment (AIMS score-13) and finally achieving a score of AIMS - 10 at the end of four weeks. He reported no side effects at the current dose of Tetrabenazine 75mg. In the next follow-up the dose of T Tetrabenazine was increased to 150 mg /day and T Clonazepam 2mg and T Vit. E 1600 mg./ day. The patient continued to show further improvement in the following visits and there were no conspicuous side effects. Our patient had the common *oral-bucco-lingual variety* of TD.[1] Being a male and age around 40 years were the points against the diagnosis of TD.[1,3,4]

Tetrabenazine, a benzoquinolizine derivative, which depletes presynaptic dopamine and serotonin storage and antagonizes postsynaptic dopamine receptors, has been used to treat TD though it has no FDA approval for the same. In a video monitored trial 20 patients of refractory tardive dyskinesia treated with Tetrabenazine (mean duration-20.3 weeks) was well tolerated in dose range of 25 mg and gradually increased upto150 mg (mean 57.9 mg /day). There was a significant improvement in AIMS scores for patients. No other drug was added in above study.[6] Similarly our patient reported significant improvement in the AIMS scores (from basal score 21 to 13 and subsequently 10) was noted when treated with a combination of tetrabinazine (used in a maximum dose of upto 150 mg) along with clonazepam and Vitamin E. The addition of clonazepam and Vitamin E to our regime could have been of advantage as both of them act synergistically by acting through the GABAnergic and free radical neuroprotective mechanisms respectively.[1,4] In conclusion, the combination used above yielded good result in a single patient and could be generalized by conducting trials in larger groups.
